# Multifluid Metabolomics Identifies Novel Biomarkers for Irritable Bowel Syndrome

**DOI:** 10.3390/metabo15020121

**Published:** 2025-02-12

**Authors:** Daniel Kirk, Panayiotis Louca, Ilias Attaye, Xinyuan Zhang, Kari E. Wong, Gregory A. Michelotti, Mario Falchi, Ana M. Valdes, Frances M. K. Williams, Cristina Menni

**Affiliations:** 1 Department of Twin Research & Genetic Epidemiology, King’s College London, London SE1 7EH, UK; daniel.1.kirk@kcl.ac.uk (D.K.); panayiotis.louca@kcl.ac.uk (P.L.); i.attaye@erasmusmc.nl (I.A.); xinyuan.zhang@kcl.ac.uk (X.Z.); mario.falchi@kcl.ac.uk (M.F.); ana.valdes@nottingham.ac.uk (A.M.V.); frances.williams@kcl.ac.uk (F.M.K.W.); 2 Amsterdam Cardiovascular Sciences, Diabetes & Metabolism, 1105 AZ Amsterdam, The Netherlands; 3 Department of Internal Medicine, Division of Nephrology and Transplantation, Erasmus MC, University Medical Center, 3015 GD Rotterdam, The Netherlands; 4 Metabolon Inc., Research Triangle Park, Morrisville, NC 27560, USA; kwong@metabolon.com (K.E.W.); gmichelotti@metabolon.com (G.A.M.); 5 Nottingham NIHR Biomedical Research Centre, School of Medicine, University of Nottingham, Nottingham NG5 1PB, UK; 6 Inflammation, Recovery and Injury Sciences, School of Medicine, University of Nottingham, Nottingham NG7 2UH, UK; 7 Department of Pathophysiology and Transplantation, Università Degli Studi di Milano, 20122 Milan, Italy

**Keywords:** irritable bowel syndrome, serum, stool, urine metabolomics

## Abstract

**Background/Objectives**: Irritable bowel syndrome (IBS) is a complex disorder affecting 10% of the global population, but the underlying mechanisms remain poorly understood. By integrating multifluid metabolomics, we aimed to identify metabolite markers of IBS in a large population-based cohort. **Methods**: We included individuals from TwinsUK with and without IBS, ascertained using the Rome III criteria, and analysed serum (232 cases, 1707 controls), urine (185 cases, 1341 controls), and stool (186 cases, 1284 controls) metabolites (Metabolon Inc.). **Results**: After adjusting for covariates, and multiple testing, 44 unique metabolites (25 novel) were associated with IBS, including lipids, amino acids, and xenobiotics. Androsterone sulphate, a sulfated steroid hormone precursor, was associated with lower odds of IBS in both urine (0.69 [95% confidence interval = 0.56–0.85], *p* = 2.34 × 10^−4^) and serum (0.75 [0.63–0.90], *p *= 1.54 × 10^−3^. Moreover, suberate (C8-DC) was associated with higher odds of IBS in serum (1.36 [1.15–1.61]; *p* = 1.84 × 10^−4^) and lower odds of IBS in stool (0.76 [0.63–0.91]; *p* = 2.30 × 10^−3^). On the contrary, 32 metabolites appeared to be fluid-specific, including indole, 13-HODE + 9-HODE, pterin, bilirubin (E,Z or Z,Z), and urolithin. The remaining 10 metabolites were associated with IBS in one fluid with suggestive evidence (*p* < 0.05) in another fluid. Finally, we identified androgenic signalling, dicarboxylates, haemoglobin, and porphyrin metabolism to be significantly over-represented in individuals with IBS compared to controls. **Conclusions**: Our results highlight the utility of a multi-fluid approach in IBS research, revealing distinct metabolic signatures across biofluids.

## 1. Introduction

Irritable bowel syndrome (IBS) is a disorder of gut–brain interaction affecting 1 in 10 people worldwide, impacting the quality of life in those affected [1]. IBS is considered a collection of symptoms that can vary amongst individuals and over time, with the most common being abdominal pain and cramps along with alterations in bowel movement, frequency, and stool consistency [1]. Around 30–50% of IBS patients have unexplained extra-gastrointestinal comorbidity such as fatigue, pain, and fibromyalgia, amongst others [2].

The pathophysiology of IBS remains poorly understood, with known risk factors including diet, genetics, medication use, stress (particularly early-life stress or trauma), and gastroenteritis infection [1,3]. Gastrointestinal changes may also involve alteration in gut smooth muscle function, visceral hypersensitivity, motility, weakened gut barrier integrity, and a disrupted gut microbiome [4,5]. Diagnosis of IBS is based on the Rome III or Rome IV criteria, which focus on the frequency of abdominal pain or discomfort and associated changes in stool frequency or consistency within a defined timeframe [6]. Based on these symptom patterns, IBS is categorised into subtypes: diarrhoea-dominant (IBS-D), constipation-dominant (IBS-C), or mixed (IBS-M) IBS [6]. However, due to overlapping symptoms with other gastrointestinal disorders, misdiagnosis is common, often resulting in delays before receiving an accurate diagnosis which is a diagnosis of exclusion. Identifying biomarkers could improve diagnostic speed and accuracy, reduce referral to secondary care, and provide insights into the mechanisms and potential therapeutic options for IBS.

In recent years, the metabolome has emerged as a promising source of biomarkers and is highly relevant to gut-based pathology [7,8,9,10]. Metabolites represent the intermediate outcomes of physiological processes and are influenced by disease, lifestyle, environmental exposure, and the gut microbiome [11]. Their dynamic nature makes them ideal for diagnostic and prognostic purposes, helping to predict and monitor treatment effectiveness [12]. Indeed, previous studies have identified IBS-related metabolites, including hypoxanthine, tryptophan, short-chain fatty acids (SCFAs), bile acids, and metabolites of bilirubin in stool [9,13,14,15]; bilirubin, carnitine, amines, fatty acids, and triglycerides in serum [16]; and 2-hydroxyglutarate, histidine, creatinine, citrate, and hydroxylysine metabolites, amongst others [17,18,19]. However, consistent and reproducible biomarkers remain elusive.

By integrating high-throughput metabolomics data from serum, urine, and stool, we aim to identify novel metabolite markers of IBS within a large population-based cohort and to uncover the underlying molecular pathways.

## 2. Materials and Methods

### 2.1. Study Cohort

We included individuals from the TwinsUK cohort, a UK-based adult twin registry consisting of over 16,000 participants with a wealth of phenotypic and biological data for studying health- and social-related questions [20]. TwinsUK study participants are predominantly middle-aged females, and there is an equal proportion of monozygotic and dizygotic pairs. In this study, we included twins who had completed the Rome III questionnaire [20] at one or both time points, in 2009 or 2016, and had metabolomic profiling (urine, serum, or stool) either within the 3 years prior to or within one year after an IBS status questionnaire response date. 

### 2.2. Irritable Bowel Syndrome Definition

Responses to questions related to the Rome III criteria were used to determine IBS status (Table S1). Individuals were classified as IBS cases if they met the Rome III criteria for IBS at either time point. Controls were defined as participants who did not meet the Rome III criteria and self-reported never having IBS on all questionnaire responses at all time points.

Participants who had not answered questions related to the Rome III criteria before their most recent metabolomic sampling date were excluded from the analysis. Additionally, participants who either met the Rome III criteria or self-reported having been diagnosed with IBS after their most recent metabolomic sampling date were also excluded.

#### IBS Subtype

Rome III criteria responses were used to distinguish participants with IBS-C, IBS-D, and IBS-M. After meeting the Rome III criteria, a participant was classified as having IBS-C if they also reported often (or more frequently) harder stools or less frequent bowel movements, and IBS-D if they also reported often (or more frequently) looser stools or more frequent bowel movements. If they qualified for both or if their subtype changed from one to the other between the two time points when the Rome III criteria were reported, they were classified as IBS-M.

### 2.3. Dietary Data

A validated 131-item semi-quantitative food frequency questionnaire (FFQ), originally developed for the European Prospective Investigation into Cancer and Nutrition (EPIC)-Norfolk study [21], was used to estimate habitual dietary intake. Food item, macronutrient, and micronutrient intakes were calculated from the FFQs using FETA software v2.53 [21,22]. We then derived the Healthy Eating Index (HEI), a cumulative score ranging from 0 to 100 points that accounts for 12 dietary components reflecting recommendations based on the dietary guidelines for Americans [23].

### 2.4. Medication Use

Drug information was derived from self-reported questionnaires on medication use, with the response closest to the date of metabolomics sampling being used to determine drugs used at the time. We included data on drugs that were potentially related to IBS from the published literature [24]. Classes of pharmaceuticals treating similar conditions were merged (see Supplementary Text). Only drugs taken by ≥1% of the sample population were retained, and then only drugs that were taken differently in participants with and without IBS, as determined by Chi-squared tests (FDR < 0.05 [25]) were kept.

### 2.5. Metabolomics

Metabolite concentrations in serum, urine, and stool were quantified by Metabolon, Inc. (Durham, NC, USA) using liquid and gas chromatography via Waters ACQUITY ultra-performance liquid chromatographer coupled with untargeted mass spectrometry via Thermo Scientific Q-Exactive interfaced with heated electrospray ionization source and Orbitrap mass analyser, as previously described [26,27]. Briefly, metabolites were quantified by the area-under-the-curve method. Data were normalised by run-day median, and inverse normalized [26]. Metabolic traits with more than 20% missing values were excluded, and missing data in the remaining metabolites were imputed using the run-day minimum [26].

The Metabolon panel includes a broad set of metabolites belonging to the following super-pathways: lipid, amino acid, xenobiotics, nucleotide, cofactor and vitamins, carbohydrate, peptide, and energy, along with those partially characterized. In each fluid, only metabolites of known chemical identity were used in the analysis.

### 2.6. Statistical Analysis

#### 2.6.1. Statistical Modelling

All analyses were performed in R statistical software (version 4.3.3). Univariate logistic mixed models were fit using the lmer package [28] to identify metabolites associated with the dependent variable IBS whilst adjusting for age, sex, and BMI as fixed effects, and family ID as a random effect and multiple testing (FDR < 0.1 [25]). Sensitivity analyses were performed, further adjusting for (i) inflammatory bowel disease (IBD) status, (ii) the HEI index and its 12 individual components, and by (iii) IBS-related medications, as described in the Supplementary Text. To determine which IBS subtype was driving the associations, for each IBS-associated metabolite, we ran two univariate models, one comparing individuals with IBS-C with healthy controls and another comparing individuals with IBS-D with healthy controls. Due to the low number of participants with IBS-M, this subtype was not included in the subtype analysis.

#### 2.6.2. Pathway Enrichment Analysis

We conducted pathway enrichment over-representation analysis [29] by comparing the observed frequency of each pathway among associated metabolites to the expected frequency by chance, using hypergeometric tests (R function “phyper” function, with *q* being the number of associated metabolites in each pathway, *m* being the number of metabolites in the same pathway in the reference dataset, *n* being the number of metabolites in the reference dataset minus m, *k* being the number of associated metabolites (i.e., 44), and “lower.tail” set to false. As the reference dataset, we used all the metabolites of known chemical identity measured by Metabolon in any of the three fluids. To avoid artificially expanding the reference database with duplicate entries across multiple fluids, if a metabolite was present in more than one fluid, only a single entry was retained.

## 3. Results

### 3.1. Descriptive Characteristics

The descriptive characteristics of the study population by fluid are reported in Table 1 and a flowchart of the number of participants at each data processing step is presented in Figure S1. Briefly, we included 185 IBS cases and 1341 controls with urinary metabolites, 232 IBS cases and 1707 controls with serum metabolites, and 186 IBS cases and 1284 controls with stool metabolites, measured within 0.61 (0.65) years. Included individuals were middle-aged (mean age 61.06–63.33 years), mainly female (85.13–87.98%), and slightly overweight (BMI = ~26 kg/m^2^). Around 10% of study participants were taking anti-depressants, 7% were on analgesics, 2% on laxatives, and 1.6% on anti-spasmodics and anti-motility drugs.

### 3.2. Multi-Fluid IBS-Metabolite Associations

**Figure 1:**
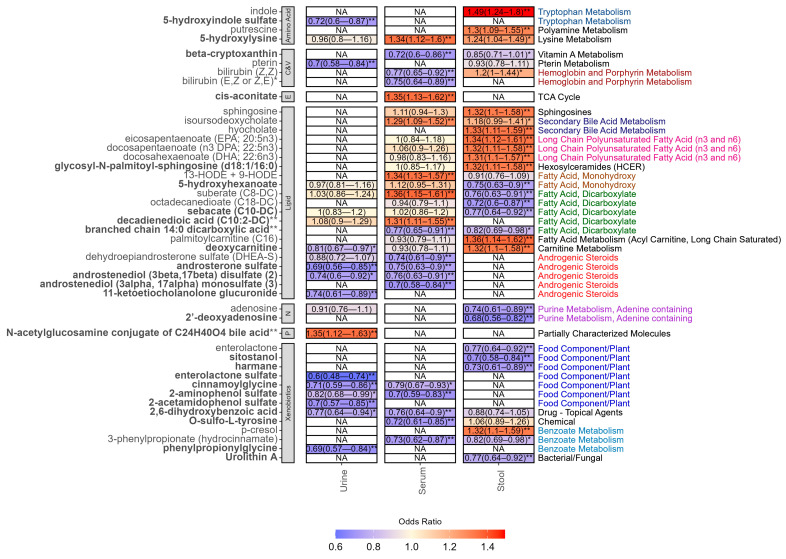
Odds ratio and 95% confidence intervals of all metabolites associated with IBS in at least one fluid after adjusting for covariates, family, and multiple testing (FDR < 0.1). Metabolite names in bold denote novel finding in IBS. NA denotes that the metabolite was not measured in the fluid. ** FDR < 0.1. * *p*-value < 0.05. Sub-pathways are depicted on the right-hand side. Each sub-pathway is colour coded. Black indicates pathways that only appear once. Abbreviations: C&V: cofactors and vitamins; E: energy; N: nucleotide.

After adjusting for covariates and multiple testing, we identified 46 unique metabolites correlated with IBS, including 21 lipids, 13 xenobiotics, 4 amino acids, 4 cofactor and vitamins, two nucleotides, one energy-related metabolite and one partially characterised molecule (Figure 1). Two metabolites, namely androsterone sulphate and suberate (C8-DC), were associated with IBS in multiple fluids (androsterone sulphate urine: OR [95%CI] = 0.69 [0.56–0.85]; *p* = 2.34 × 10^−4^); serum: 0.75 [0.63–0.90]; *p* = 1.54 × 10^−3^; suberate (C8-DC) serum: 1.36 [1.15–1.61]; *p* = 1.84 × 10^−4^; stool: 0.76 [0.63–0.91]; *p* = 2.30 × 10^−3^). The remaining metabolites were either fluid-specific (*n* = 33) or were nominally associated (*p* < 0.05) in more than one fluid. Importantly, concordant across-fluid effects were observed for nine of the metabolites associated in multiple fluids, including 5-hydroxylysine, which was positively correlated with higher odds of IBS in both serum and stool (serum: 1.34 [1.12–1.60]; *p* = 9.13 × 10^−3^; stool: 1.24 [1.04–1.49]; *p* = 0.02) and 3-phenylpropionate (hydrocinnamate), which displayed negative correlations in serum and stool (serum: 0.73 [0.62–0.87]; *p* = 1.92 × 10^−4^; stool: 0.82 [0.69–0.98]; *p* = 0.03). In contrast, opposing patterns were observed for both bilirubin (Z,Z) (serum: 0.77 [0.65–0.92]; *p* = 2.21 × 10^−3^; stool: 1.20 [1.00–1.44]; *p* = 0.04) and deoxycarnitine (stool: 1.32 [1.10–1.58]; *p* = 2.12 × 10^−3^; urine: 0.81 [0.67–0.97]; *p* = 0.02).

### 3.3. Fluid-Specific Associations

#### 3.3.1. Urine Metabolites

As displayed in Figure 1, all but one of the seven IBS-associated urine-specific metabolites were all lower in individuals with IBS compared to controls, the exception being a partially characterised bile acid conjugate (N-acetylglucosamine conjugate of C_24_H_40_O_4_ bile acid **, 1.35 [1.12–1.63]; *p* = 1.13 × 10^−3^). ORs in the remaining urine metabolites ranged from 0.60 (0.48–0.74; *p* = 1.10 × 10^−7^) for enterolactone sulphate to 0.74 (0.61–0.89; *p* = 1.00 × 10^−3^) for 11-ketoetiocholanolone glucuronide.

#### 3.3.2. Serum Metabolites

We identified eight serum-specific metabolites associated with IBS. Three of these, were higher in IBS compared to controls, namely HODE 9 + 13 (1.34 [1.13–1.57]; *p* = 3.82 × 10^−4^), cis-aconitate (1.35 [1.13–1.62]; *p* = 5.71 × 10^−4^), and decadienedioic acid (C10:2-DC) ** (1.31 [1.11–1.55]; *p* = 1.17 × 10^−3^), the latter two of which are novel findings. Serum metabolites that were lower in individuals with IBS compared to controls included androstenediol (3alpha, 17alpha) monosulphate (3) (0.70 [0.58–0.84]; *p* = 1.03 × 10^−4^), O-sulfo-L-tyrosine (0.72 [0.61–0.85; *p* = 5.81 × 10^−5^), and bilirubin (E,Z or Z,E) * (0.75 [0.64–0.89]; *p* = 8.05 × 10^−4^), amongst others.

#### 3.3.3. Stool Metabolites

In total, 19 metabolites were associated with IBS in stool only. Negative associations were observed for the nucleotide 2′-deoxyadenosine (0.68 [0.56–0.82]; *p* = 2.04 × 10^−5^), phytosterol sitostanol (0.70 [0.58–0.84]; *p* = 8.03 × 10^−5^), and three dicarboxylates. The remaining ten metabolites were higher in IBS cases compared to controls. The strongest association was seen for indole, (1.49 [1.24–1.80]; *p* = 7.83 × 10^−6^), with others including palmitoylcarnitine (C16) (1.36 [1.14–1.62]; *p* = 5.56 × 10^−4^), the bile acid hyocholate (1.33 [1.11–1.59]; *p* = 1.35 × 10^−3^), and *p*-cresol (1.32 [1.10–1.59]; *p* = 1.78 × 10^−3^).

### 3.4. Sensitivity Analysis

As we have previously reported proton pump inhibitors (PPI) to correlate with metabolites involved in androgenic signalling [30], we reran the analysis for the IBS-associated metabolites, further adjusting for PPI usage and the results remained consistent. We also ran sensitivity analysis further adjusting for IBD status, IBS-related medications (including anti-spasmodic and anti-motility drugs, anti-depressant and anxiolytics, laxatives, and analgesics) and for diet, measured by the HEI index and by its individual components. The results remained consistent (Figure S2), except for androstenediol (3beta,17beta) disulphate (2), which was no longer significant after adjusting for medication, and the stool metabolite docosapentaenoate (n3 DPA; 22:5n3), which was no longer significant after adjusting for HEI components. Associations were weaker for indole, androgenic steroids, and *p*-cresol after adjusting for medication, and for beta-cryptoxanthin, sphingosine, hyocholate, deoxycarnitine, and various fatty acids after adjusting for diet.

### 3.5. Subtype Analysis

**Figure 2:**
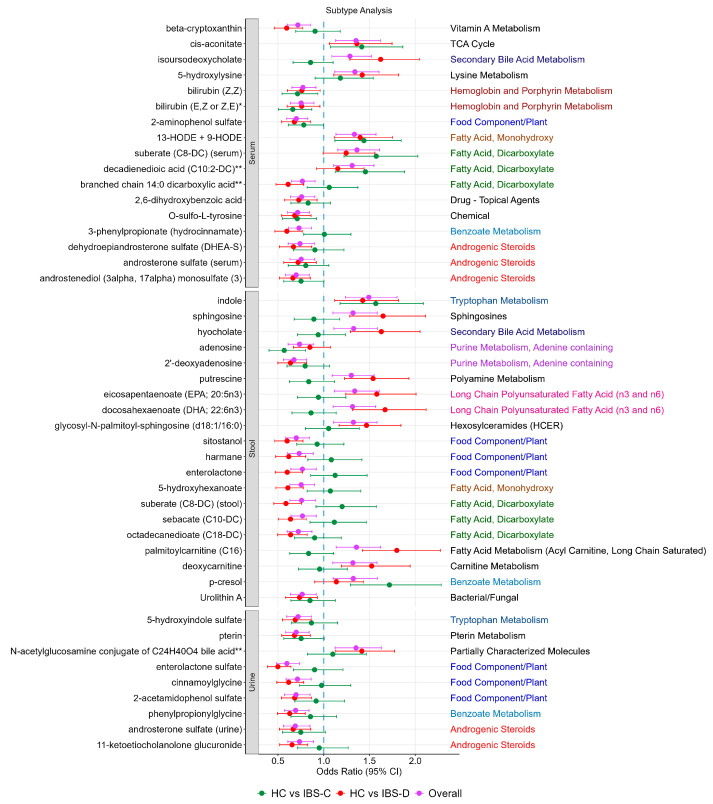
IBS-associated metabolites and their association stratified by IBS subtype. For each of the metabolites, sub-pathways are depicted on the right-hand side. Each sub-pathway is colour coded. Black indicates pathways that only appear once.

For the IBS-associated metabolites, we rerun the analysis comparing healthy controls to each subgroup (IBS-C or IBS-D). The results suggested that some of the associations appeared to be driven by one subtype in particular (Figure 2). Those that appeared to be mainly driven by IBS-C included the dicarboxylic acids decadienedioic acid (C10:2-DC) **, suberate (C8-DC) (in serum only), adenosine, and *p*-cresol, whereas those driven by IBS-D were enterlactone sulphate, palmitoylcarnitine (C16), deoxycarnitine, putrescine isoursodeoxycholate, hyocholate, sphingosine, branched chain 14:0 dicarboxylic acid **, DHEA-S, and the dicarboxylates and omega-3 fatty acids in stool.

### 3.6. Enrichment Analysis

**Figure 3:**
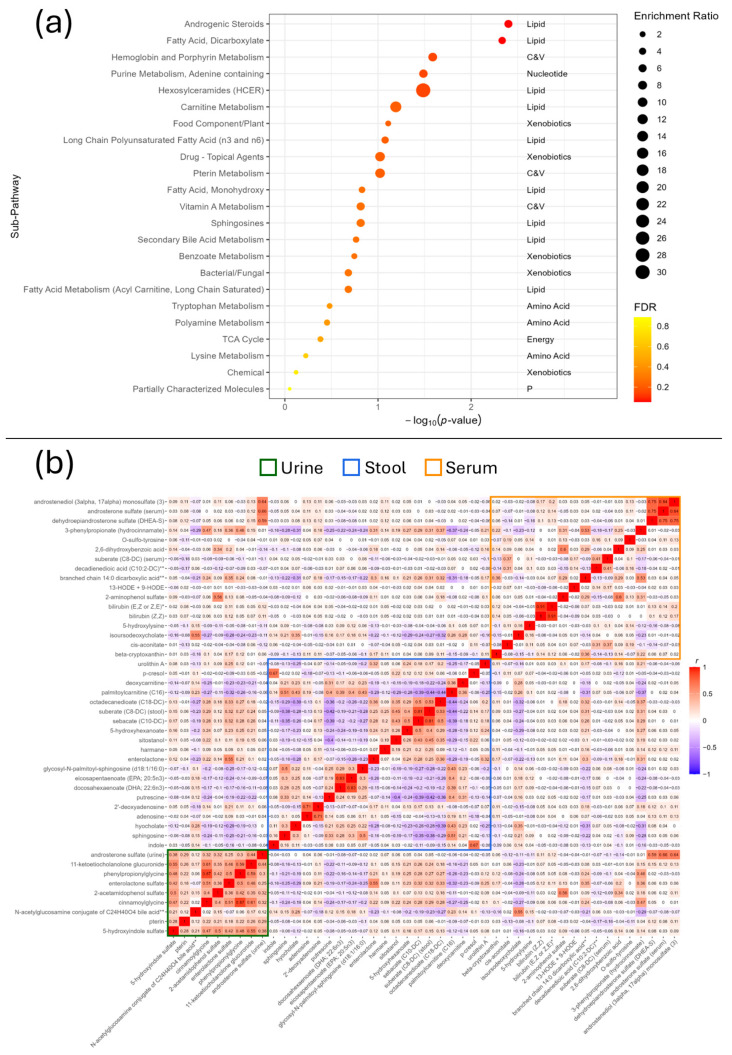
(**a**) Enrichment analysis of the IBS-associated metabolites. Abbreviations: C&V: cofactors and vitamins; *p*: partially characterised molecules. (**b**) Heatmap of the Pearson correlations between the IBS-associated metabolites across all fluids. Serum is represented by the orange rectangle, stool the blue, and urine the green.

Enrichment analysis identified over-representation of androgenic steroids (enrichment ratio [ER] = 5.82, FDR = 0.05) and dicarboxylates (ER = 4.37, FDR = 0.05). Nominally associated (*p* < 0.05) pathways included haemoglobin and porphyrin metabolism (ER = 7.64, *p* = 0.03), adenine-containing purine metabolism (ER = 6.79, *p* = 0.03), and hexosylceramides (HCER) (ER = 30.57, *p* = 0.03), which contained only one metabolite (glycosyl-N-palmitoyl-sphingosine (d18:1/16:0)) (Figure 3a).

To further assess the enrichment of key pathways, the Pearson’s correlation of all IBS-associated metabolites was also calculated and plotted which identified important groups of correlated variables within each fluid (Figure 3b). Key examples of this include the androgenic steroids androstenediol (3alpha, 17alpha) monosulphate (3), and dehydroepiandrosterone sulphate (DHEA-S) in serum and androsterone sulphate in both urine and serum; a positive correlation between indole and *p*-cresol in stool; 2,6 dihydroxybenzoic acid and 2-aminophenol sulphate in serum; and positive correlations between the urine metabolites in general, though particularly between cinnamoylglycine, phenylpropionylglycine, and 11-ketoetiocholanolone glucuronide. The two isomers of bilirubin were also highly correlated (*r* = 0.91).

## 4. Discussion

**Figure 4:**
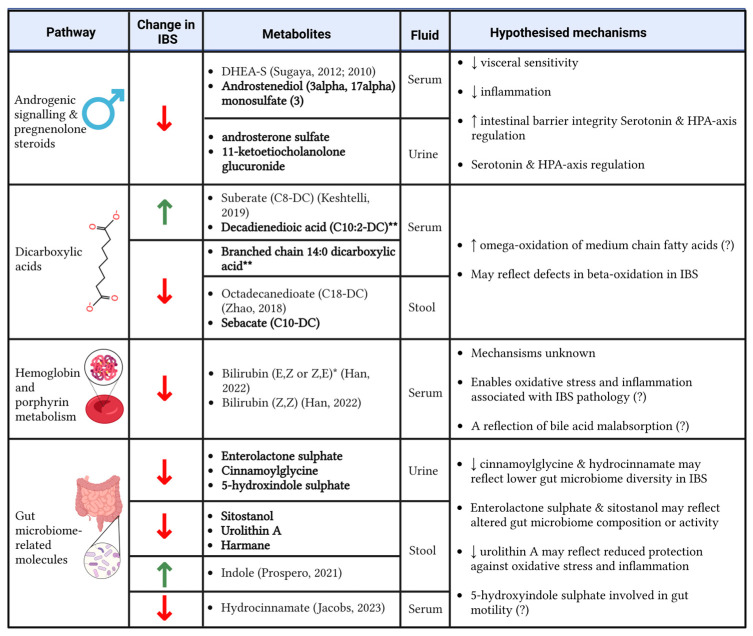
Significant enriched pathways and metabolites that were altered in individuals with IBS compared to healthy controls and the potential mechanisms driving the associations. Bold metabolite names denote novel findings; if the metabolite association with IBS is not novel, references are provided [9,16,31,32,33,34,44]. (?) denotes a lower degree of certainty around the hypothesised mechanism. Created using BioRender. Menni, C. (2025) https://BioRender.com/v90g765.

In the first study to use urine, serum, and stool samples simultaneously to investigate metabolite alterations in IBS, we identified 44 metabolite biomarkers of IBS. As reported in Figure 4, some of these biomarkers have been previously linked to IBS [8,9,14,16,31,32,33,34,35,36,37,38,39,40] (Table S2), underscoring the robustness of our results. Importantly, however, 25 metabolites appear to be novel. These include cis-aconitate, which has been previously reported to correlate with inflammation and disease activity in children with Crohn’s disease [41]; 2′-deoxyadenosine, a nucleotide associated with colorectal cancer in organoids [42]; and deoxycarnitine, which has been linked gut permeability [43]. This opens up new avenues for understanding the mechanisms of IBS and their potential overlap with other gastrointestinal diseases.

Our findings also reveal that most metabolite associations and the sub-pathways they belong to are fluid-specific. This is important as though the fluids are related to one another through processes of metabolism and excretion [45], with urine being a filtrate of serum and many serum metabolites being derived from the gut [46], each fluid provides unique information reflecting the underlying metabolic processes. This highlights the importance of multi-fluid metabolomics approaches to identify disease biomarkers. Furthermore, our results suggest that some associations may be driven by certain IBS subtypes, meaning that some metabolomic alterations may be subtype-specific.

### 4.1. Metabolites Associated in Multiple Fluids

Androsterone sulphate and suberate (C8-DC) were the only metabolites that passed multiple testing correction in more than one biofluid (urine and serum and serum and stool, respectively). We also identified 10 metabolites that correlated with IBS after adjusting for multiple testing in one fluid but with suggestive evidence in another fluid (*p* < 0.05). To the best of our knowledge, 7 of these are novel findings, namely cinnamoylglycine, 5-hydroxylysine, branched chain 14:0 dicarboxylic acid **, 2-aminophenol sulphate, 2,6-dihydroxybenzoic acid, deoxycarnitine, and the vitamin A metabolite beta-cryptoxanthin [47].

Cinnamoylglycine, which was negatively associated with IBS in urine (after FDR adjustment) and serum (nominal), has been previously associated with lower gut microbiome diversity [48], which is known to correlate with IBS in some studies [49]. Elevated 5-hydroxylisine may suggest enhanced collagen degradation [50], potentially driven by low-grade inflammation-mediated acceleration of collagen degradation and epithelial turnover in IBS [18]. Interestingly, suberate, which was elevated in the serum and decreased in the stool of participants with IBS, has been shown to promote collagen synthesis in pre-clinical skin studies [51]. The remaining three metabolites which were FDR-associated in one fluid and nominally associated in another have been previously described in IBS, namely 3-phenylpropionate (hydrocinnamate) [9], isoursodeoxycholate [9], and bilirubin (Z,Z) [16].

### 4.2. Tissue-Specific Associations

The remaining 32 metabolites were associated with IBS in a single fluid, including 14 lipids, 9 xenobiotics, 3 amino acids, 2 cofactors and vitamins, 2 nucleotides,1 metabolite related to energy, and 1 partially characterised molecule. To the best of our knowledge, 17 of these metabolites have never been reported in IBS, with several relating to the diet and gut microbiome composition and activity (enterolactone sulphate, sitostanol, urolithin A, and harmane), immunomodulation (5-hydroxyhexanoate [52]), bile acid metabolism (*N*-acetylglucosamine conjugate of C_24_H_40_O_4_ bile acid **), and hexosylceramides (glycosyl-*N*-palmitoyl-sphingosine (d18:1/16:0)).

Importantly, while we did not find evidence for alterations in neurotransmitters thought to be involved in IBS, such as serotonin [53], related metabolites 5-hydroxyindole sulphate and indole were associated with lower odds of IBS in urine and higher odds in stool, respectively. Similarly, while we found no alteration in tyrosine, an amino acid precursor to neurotransmitters dopamine and norepinephrine, O-sulfo-l-tyrosine was associated with lower odds of IBS in serum. Both 5-hydroxyindole sulphate and O-sulfo-l-tyrosine also represent novel tissue-specific findings in IBS.

The remaining tissue-specific metabolites have been previously implicated in IBS, with examples including bilirubin (E,Z or Z,E) * and DHEA-S in serum and *p*-cresol, adenosine, and octadecanedioate (C18-DC) in stool.

Higher levels of faecal adenosine have been reported in individuals with IBS compared to healthy controls in a large-scale study [8]. Additionally, the association between adenosine and IBS has been proposed due to its role in key physiological processes, including inflammation, visceral pain, and sensory functions such as regulating gut motility in the large intestine [54]. DHEA-S is another molecule involved in androgen signalling and was decreased in the serum overall and in the subtype analysis, where its association was shown to be driven by IBS-D. It has been previously observed depleted in the saliva of college students with IBS [34]. Finally, octadecanedioic acid was lower in stool samples in IBS compared to controls in a study involving 357 individuals with IBS and 84 controls [33].

Enrichment analysis identified significant pathways in IBS, including androgenic signalling, dicarboxylates, haemoglobin and porphyrin metabolism, adenine-containing purine metabolism, and hexosylceramides. As described in Figure 4, we have hypothesised the underlying mechanisms that could link some of these pathways to IBS.

### 4.3. Androgen Signalling

Four of the metabolites that differed between IBS and healthy controls were related to androgen signalling, including DHEA-S, androstenediol (3alpha, 17alpha) monosulphate (3), androsterone sulphate, and 11-ketoetiocholanolone glucuronide These metabolites were correlated with each other (Figure 3b), lower in IBS compared to controls, and all but one were novel findings (DHEA-S being the exception [34]).

Alteration in sex hormone levels has been previously described with IBS and has been hypothesised to contribute to sex-based differences in IBS prevalence, with higher rates observed in females compared to males [55]. Consistent with our findings, it has been suggested that androgens may exert a protective effect against IBS [56]. The proposed mechanisms underlying this effect include reduced visceral sensitivity, enhanced integrity of the intestinal barrier integrity, modulation of serotonin signalling, regulation of the hypothalamus–pituitary axis regulation, and reduced inflammation; however, some of these mechanisms may exhibit sex-specific variability [55]. Of the metabolites in this group that we identified, these properties have been displayed for DHEA-S, which prevented visceral allodynia and improved gut barrier function in a rat model of IBS [57]. We have also reported a link between DHEA-S and chronic widespread pain [58]. Both IBS and chronic widespread pain form a part of genetically linked chronic pain syndromes, and thus DHEA-S may represent a common metabolite shared by both conditions that is involved in nociception [59].

As for the other metabolites in this pathway, it is likely that their reductions reflect lower levels of androgens which may be more directly involved in IBS symptomology, such as testosterone, DHEA-S, and dihydrotestosterone, rather than playing a causal role themselves. It is also possible that medication might explain some of the variance in the associations we observed. Possible candidates for this include the group of analgesics, particularly opioid analgesics, which may be taken by individuals with IBS and pain comorbidity and can induce androgen deficiency through HPA-axis suppression [60]. However, sensitivity analyses adjusting for use of medication, including opioids, find similar results, suggesting that this is not a major factor and does not alter our conclusions.

### 4.4. Fatty Acids

Alterations in certain lipids may suggest defects in fatty acid oxidation in IBS. Five dicarboxylates were altered in IBS: suberate (C8-DC), decadienedioic acid (C10:2-DC) ** and branched chain 14:0 dicarboxylic acid ** in serum, and octadecanedioate (C18-DC) and sebacate (C10-DC) in stool. Decadienedioic acid (C10:2-DC) **, sebacate (C10-DC), and branched chain 14:0 dicarboxylic acid ** are novel findings for IBS, whereas octadecanedioate has been previously reported elsewhere to be lower in IBS [33] and suberate was lower in the urine of individuals with IBS-M compared to IBS-D [32].

Dicarboxylic acids are products of omega-oxidation, an alternative pathway for oxidising fatty acids when beta-oxidation is impaired [61]. In our data, circulating levels of both suberate (C8-DC) and decadienedioic acid (C10:2-DC) ** were correlated with higher odds of IBS, suggesting that an increase in omega-oxidation could be a compensatory mechanism for defects in beta-oxidation in IBS. These defects would appear to be limited to medium-chain fatty acids, given that serum branched chain 14:0 dicarboxylic acid ** and octadecanedioate (C18-DC) were lower and unchanged, respectively, in IBS. Interestingly, these changes may be partially dependent on the IBS subtypes, as the increases in serum suberate (C8-DC) and decadienedioic acid (C10:2-DC) ** were mostly driven by IBS-C, whereas decreased 14:0 dicarboxylic acid ** in serum and sebacate (C10-DC) and octadecanedioate (C18-DC) in the stool were driven by IBS-D. This is consistent with previous work suggesting IBS is linked to defects in beta-oxidation [8,62] or energy metabolism [13,63], and alterations in other dicarboxylates [16,64].

Previous studies have reported differences in polyunsaturated fatty acids (PUFA) in IBS [35,36,65]. More recently, a small study observed decreases in PUFAs in the colonic mucosa of IBS-D patients compared to controls. Such reduction in mucosal PUFAs may to be due to increased losses via stool, potentially diminishing their anti-inflammatory effects in the large intestine and exacerbating symptoms [66]. In our own data, we observed higher faecal abundance of eicosapentaenoate (EPA; 20:5n3), docosahexaenoate (DHA; 22:6n3), and docosapentaenoate (n3 DPA; 22:5n3) in individuals with IBS compared to controls. However, after adjusting for dietary intake, we found no significant difference in docosapentaenoate (n3 DPA; 22:5n3).

Additionally, whilst short-chain fatty acids (SCFAs) are often postulated to be involved in IBS [67], we did not identify any metabolites related to SCFA metabolism in the analyses.

Finally, we expected to observe links between endocannabinoid-related compounds and IBS, given the strong literature on this topic which shows that the endocannabinoid system regulates GI motility, secretion, barrier function, inflammation, and visceral sensitivity [68]. However, none of the endocannabinoid compounds in the panel showed an association in any of the tissues tested with IBS.

### 4.5. Haemoglobin and Porphyrin Metabolism

Two isomers of bilirubin were depleted in serum, highlighting alterations in haemoglobin and porphyrin metabolism pathways. Alterations in bilirubin and metabolites thereof in IBS have been reported in multiple other studies [8,9,16].

One proposed mechanism linking bilirubin with IBS is through oxidative stress [69]. Since bilirubin is a reactive oxygen species scavenger and serves as an antioxidant, a reduction in serum bilirubin may promote a pro-oxidative environment, which has been implicated in IBS pathology [70]. In addition to bilirubin, we also found a reduction in urinary pterin, a cofactor protective against oxidative stress and immunomodulatory [71], and urolithin A, a gut microbiome-derived metabolite with anti-inflammatory and antioxidant effects, in the stool [72]. Finally, bilirubin is reported to have immunomodulatory properties [69], meaning that a reduction in serum bilirubin could contribute to low-grade inflammation, which has been reported in IBS [73].

Our study has several strengths, including a large accurately phenotyped cohort with metabolomics profiling available across three different fluids. IBS status was determined using the Rome III criteria, though in addition to this, controls were also required to have never reported having on self-reported IBS questions. We also examined IBS subtypes to capitalise on the unique information provided by each, enhancing our understanding of the metabolic differences associated with the condition

We also note some limitations. First, the cross-sectional and observational nature of this study prevents us from determining causality between metabolites and IBS. Second, the number of participants varies across the fluids because serum samples have been collected more frequently over the years compared to stool or urine. Third, IBS status was determined via the Rome III criteria as questions for determining IBS status according to the Rome IV criteria are not available in TwinsUK [74]. Fourth, dietary data were missing for up to 17.56% of the sample and so the sensitivity analysis adjusting for diet was conducted on a smaller subset of participants dataset. Finally, we lacked a replication cohort, and further studies are needed to validate our results.

## 5. Conclusions

Using a multi-fluids approach in a large, nationally representative UK-based study sample, we have identified 46 associations from 44 metabolites correlated with IBS, including 9 in urine, 17 in serum, and 20 in stool. Most of these associations were largely unaffected following the addition of diet and medication data. Notable alterations in metabolites related to androgen signalling, dicarboxylic and omega-3 fatty acids, bilirubin, and gut microbiome-related metabolites were seen. Some of these associations appeared to be driven by one subtype, possibly suggesting different pathological mechanisms between subtypes within IBS. Future work should look to understand how these metabolites may be related to IBS, particularly those which are currently poorly characterised.

## Figures and Tables

**Table 1:** Descriptive characteristics per fluid of the TwinsUK dataset.

	Urine (*n* = 1526)		Serum (*n* = 1939)		Stool (*n* = 1470)	
	IBS Cases	Controls	IBS Cases	Controls	IBS Cases	Controls
N	185	1341	232	1707	186	1284
N females (%)	169 (91.35%)	1144 (85.31%)	212 (91.38%)	1494 (87.52%)	167 (89.78%)	1087 (84.66%)
IBS-C, *n* (%)	57(3.74%)		69 (3.65%)		59 (4.01%)	
IBS-D, *n* (%)	113 (7.40%)		137 (7.07%)		108 (7.35%)	
IBS-M, *n* (%)	15 (0.98%)		26 (1.34%)		19 (1.30%)	
IBD, *n* (%)	11 (5.95%)	16 (1.19%)	15 (6.47%)	20 (1.16%)	10 (5.38%)	17 (1.32%)
Age, years mean (SD)	61.52 (11.77)	61.00 (13.19)	62.88 (12.22)	63.39 (12.35)	62.23 (12.56)	62.77 (12.98)
BMI, kg/m^2^ mean (SD)	26.60 (5.11)	26.01 (4.96)	26.59 (5.01)	25.98 (4.89)	26.31 (4.66)	25.89 (4.84)
Anti-depressants and anxiolytics *n* (%)	35 (18.92%)	105 (7.83%)	41 (17.67%)	150 (8.79%)	35 (18.82%)	109 (8.49%)
Laxatives	12 (6.49%)	21 (1.57%)	14 (6.03%)	20 (1.17%)	12 (6.45%)	20 (1.56%)
Antispasmodics and antimotility *n* (%)	13 (7.03%)	10 (0.75%)	17 (7.33%)	15 (0.88%)	13 (6.99%)	11 (0.86%)
Analgesics, *n* (%)	25 (13.51%)	80 (5.97%)	36 (15.52%)	112 (6.56%)	27 (14.52%)	85 (6.62%)
Healthy Eating Index	59.23 (11.23)	60.02 (9.72)	59.15 (10.96)	60.34 (9.79)	58.94 (10.8)	60.12 (9.65)

## Data Availability

The data used in this study are held by the Department of Twin Research at King’s College London. The data can be released to bona fide researchers using our normal procedures overseen by the Wellcome Trust and its guidelines as part of our core funding (https://twinsuk.ac.uk/resources-for-researchers/access-our-data (accessed date 17 January 2025)).

## Supplementary Materials

The following supporting information can be downloaded at: https://www.mdpi.com/article/10.3390/metabo15020121/s1, Supplementary Text. Inflammatory bowel disease, metabolomics methodology and drug-class processing. Table S1: Questions related to the Rome III criteria in TwinsUK used to determine IBS status. To meet the Rome III criteria, participants must have had abdominal pain at least one day a week in the previous 3 months and respond “Often” or more frequently to any of the two following question categories. Table S2: List of IBS-associated metabolites previously reported to correlate with IBS. Figure S1: The key processing steps of the metabolomics datasets in each fluid, including the number of participants excluded. Figure S2: Sensitivity analysis results of IBS associated metabolites comparing the overall results (purple) with those after further adjusting for drugs (blue), HEI (orange), and food groups (green) The text on the right-hand side denotes the sub-pathway of each metabolite. Sub-pathways are colour coded, except for black, which denotes sub-pathways which only appeared once. References [75,76] are cited in the Supplementary Materials.
