# Supplementary material for: Multifluid Metabolomics Identifies Novel Biomarkers for Irritable Bowel Syndrome
Source: Metabolites. 2025 Feb 12;15(2):121. doi: 10.3390/metabo15020121 (PMC11857683; doi:10.3390/metabo15020121)

Supplementary Text

Inflammatory bowel disease

Inflammatory bowel disease (IBD) status was inferred using self-reported data. Participants who reported having been diagnosed with IBD, ulcerative colitis, or Crohn's disease on at least one occasion were investigated more closely. Participants who reported being diagnosed with one of these conditions only once across multiple dates (>3) for which data were available (and this date was not the most recent data entry) were presumed not to have mistaken entries and were assumed not to have IBD.

Metabolomics methodology

Columns and detectors

Samples were analysed using four columns: (i) Acidic positive ion conditions (hydrophilic compounds): C18 column with (Waters UPLC BEH C18-2.1×100 mm, 1.7 μm) using water and methanol, containing 0.05% perfluoropentanoic acid (PFPA) and 0.1% formic acid (FA); (ii) Acidic positive ion conditions (hydrophobic compounds): C18 column using methanol, acetonitrile, water, 0.05% PFPA, and 0.01% FA and was operated at an overall higher organic content; (iii) Basic negative ion optimised conditions: C18 column (separate from those mentioned above), where basic extracts were gradient eluted from the column using methanol and water with 6.5mM ammonium bicarbonate at pH 8. ; and (iv) Negative ionisation: HILIC column (Waters UPLC BEH amide 2.1×150 mm, 1.7 μm) using a gradient consisting of water and acetonitrile with 10mM ammonium formate, pH 10.8. Thermo Fisher Scientific Orbitrap HRAM mass spectrometers were used for detection of analytes [27].

Standards

Metabolon includes: a recovery standard, to assess the variability and verify the performance of extraction and instrumentation; and an internal standard, to assess the variability and performance of instruments. Values for instrument and process variability met Metabolon's acceptance criteria, which was for internal standards was 8% in urine, 8% for serum and 5% for stool.

An additional cocktail of QC/controls were also added to be analysed alongside the experimental samples. This included: (i) a large pool of human plasma maintained by Metabolon that has been characterized extensively to ensure that processes are operating within specifications; (ii) a pooled sample consisting of a small quantity of each experiment sample to the effect of a non-plasma matrix on the Metabolon process and distinguish biological variability from process variability; (iii) a process blank consisting of ultra-pure water to assess the contribution to compound signals from the process; and (iv) an aliquot of solvents used during extraction to account for contamination introduced during the extraction process.

Metabolite identification

Compounds were identified by comparison to library entries of purified standards or recurrent unknown entries in the Metabolon library. Detected molecules were aligned to those in this library based on retention time/index, mass to charge ratio, and chromatographic data (including MS/MS spectral data). Subsequent biochemical identifications were based on three criteria: retention index within a narrow RI window of the proposed identification, accurate mass match to the library ± 10 ppm, and the MS/MS forward and reverse scores between the experimental data and authentic standards. Metabolon can detect 1,274 metabolites in stool (1073 of known chemical identity), 1,140 metabolites in serum (970 of known chemical identity), and 1476 metabolites in urine (1044 of known chemical

identity). The great majority of those identified are level 1. Specifically, identifications are based on retention time, accurate mass measurement, and accurate mass MS/MS spectrum match to a proprietary library of authentic standards. Standards for metabolite reporting can be found here [73,74].

Drug-class processing

Medication usage was assessed in TwinsUK study participants using questionnaires as previously described [30]. For the sensitivity analysis, we considered drugs taken by more than 1% of the study sample and linked to IBS treatment [24]. These included:

- Tricyclic and related antidepressant drugs
- Antispasmodics and other drugs for altered gut motility
- Antimotility drugs
- Selective serotonin re-uptake inhibitors
- Other drugs used in constipation
- Non-opioid analgesics and compound preparations
- Opioid analgesics
- Drugs affecting biliary composition and flow
- Drugs used in nausea and vertigo
- Bulk-forming laxatives
- Soothing haemorrhoidal preparations
- Stimulant laxatives
- Osmotic laxatives
- Anxiolytics
- Thyroid hormones
- Oestrogens and HRT
- Male sex hormones and antagonists
- Parenteral progestogen-only contraceptives
- Oral progestogen-only contraceptives
- Replacement therapy
- Combined hormonal contraceptives
- Progestogens and progesterone receptor modulators

We first created the following drug classes:

- **Antidepressant and anxiolytics:** Tricyclic and related antidepressant drugs, selective serotonin re-uptake inhibitors and anxiolytics
- **Antispasmodics and antimotility**: Antispasmodics and other drugs for altered gut motility, and antimotility drugs
- **Laxatives:** Stimulant laxatives, osmotic laxatives, bulk-forming laxatives, and other drugs used in constipation
- **Analgesics:** Non-opioid analgesics and compound preparations and opioid analgesics
- **Progestogens:** Progestogens, progestogens and progesterone receptor modulators, oral progestogen-only contraceptives, and parenteral progestogen-only Contraceptives
- **Hormone replacement**: Oestrogens and HRT and replacement therapy
- **Drugs affecting biliary composition and flow**
- **Drugs used in nausea and vertigo**
- **Soothing haemorrhoidal preparations**
- **Stimulant laxatives**
- **Male sex hormones and antagonists**

- **Oral antibiotics:** including Amoxicillin, Ciprofloxacin, Clarithromycin, Cefalexin, Penicillin-V, Azithromycin, Phenoxymethylpenicillin, Fusidic Acid, Amoxil, Augmentin, Jenacillin, Erythromycin, Metronidazole, Keflex, Flagyl, Ciprofloxacin Lactate, Phenoxymethylpenicillin (Penicillin V) Flucloxacillin Sodium, Co-Amoxiclav (Amoxicillin/Clavul Acid), Colistin Sulfate, Amoxicillin Sodium.

For each drug class, we used Chi-squared tests to determine whether drug usage differed (FDR < 0.05) between individuals with IBS and controls. Individuals with IBS had significant higher usage of anti-depressants and anxiolytics, antispasmodics and antimotility drugs, analgesics, and laxatives, and so we adjusted for these four drug classes in our sensitivity analysis.

Table S1: Questions related to the Rome III criteria in TwinsUK used to determine IBS status. To meet the Rome III criteria, participants must have had abdominal pain at least one day a week in the previous 3 months and respond "Often" or more frequently to any of the two following question categories.

Question category	Question	Response options
**Abdominal pain**	In the last 3 months, how often did you have discomfort or pain anywhere in your abdomen?	0: Never<br>1: Less than one day a month<br>2: One day a month<br>3: Two or three days a month<br>4: One day a week<br>5: More than one day a week<br>6: Every day
Response "Often" or more (≥2) to any two of the following:		
**Improvement following defection**	How often did this discomfort or pain get better or stop after you had a bowel movement?	0: Never/rarely<br>1: Sometimes<br>2: Often<br>3: Most of the time<br>4: Always
**Change in bowel movement frequency**	When this discomfort or pain started, did you have more frequent bowel movements?<br><br>Or<br><br>When this discomfort or pain started, did you have less frequent bowel movements?	0: Never/rarely<br>1: Sometimes<br>2: Often<br>3: Most of the time<br>4: Always
**Change in stool consistency**	When this discomfort or pain started, were your stools (bowel movements) looser?<br><br>Or<br><br>When this discomfort or pain started, how often did you have harder stools?	0: Never/rarely<br>1: Sometimes<br>2: Often<br>3: Most of the time<br>4: Always

Table S2: List of IBS associated metabolites previously reported to correlate with IBS

Metabolite	Findings	Fluid	Reference
Indole	↑ indole in the high somatisation group versus the low somatisation group in a sample of IBS-D participants	Urine	[31]
Bilirubin (E,Z or Z,E)* and bilirubin (Z,Z)*	↑ in IBS compared to healthy controls	Serum	[9,16]
Suberate (C8-DC)	↓ in IBS-M compared to IBS-D	Serum	[32]
Docosahexaenoate (DHA; 22:6n3)	↓ in IBS compared to healthy controls	Plasma; Serum	[35,36]
Sphingosine	↓ in IBS-D compared to healthy control	Serum	[37]
Eicosapentaenoate (EPA; 20:5n3)	↓ in IBS compared to health controls	Serum	[36]
Dehydroepiandrosterone sulfate (DHEA-S)	↓ in IBS compared to healthy controls	Saliva	[34,44]
Octadecanedioate (C18-DC)	↓ in IBS compared to healthy controls	Stool	[33]
3-phenylpropionate (hydrocinnamate)	↑ in IBS compared to healthy controls	Stool	[9]
Isoursodeoxycholate	↑ in IBS-D compared to IBS-C	Stool	[9]
p-cresol	↓ in IBS-D compared to IBS-C	Stool	[9]
Enterolactone	↓ in IBS-D compared to IBS-C	Stool	[9]
Pterin	↓ in IBS-D compared to IBS-C & ↑ in IBS compared to healthy controls in females	Stool	[9,38]
Putrescine	↑ in IBS-D compared to healthy controls	Stool	[39]
Palmitoylcarnitine (C16)	↑ in IBS-D compared to healthy controls	Stool	[9]
Adenosine	↑ in IBS compared to healthy controls	Stool	[8]
Hyocholate	↑ in IBS compared to healthy control and correlated with abdominal pain in children	Stool	[40]
13-HODE + 9-HODE	↑ in IBS compared to healthy controls in females	Stool	[38]

Figure S1. The key processing steps of the metabolomics datasets in each fluid, including the number of participants excluded.

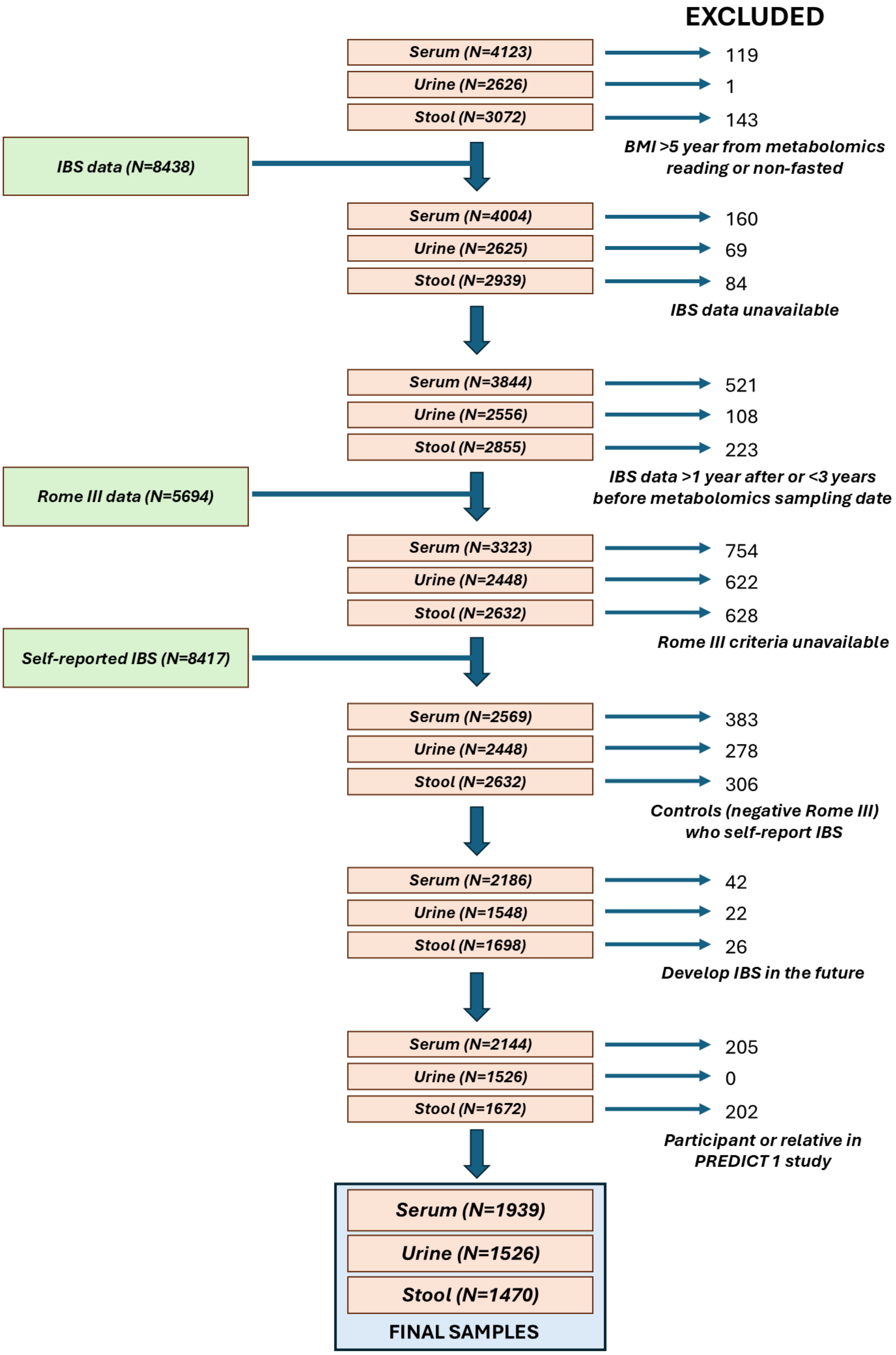

Figure S2. Sensitivity analysis results of IBS-associated metabolites comparing the overall results (purple) with those after further adjusting for drugs (blue), HEI (green), and food groups (orange). The text on the right-hand side denotes the sub-pathway of each metabolite. Sub-pathways are colour coded, except for black, which denotes sub-pathways which only appeared once.

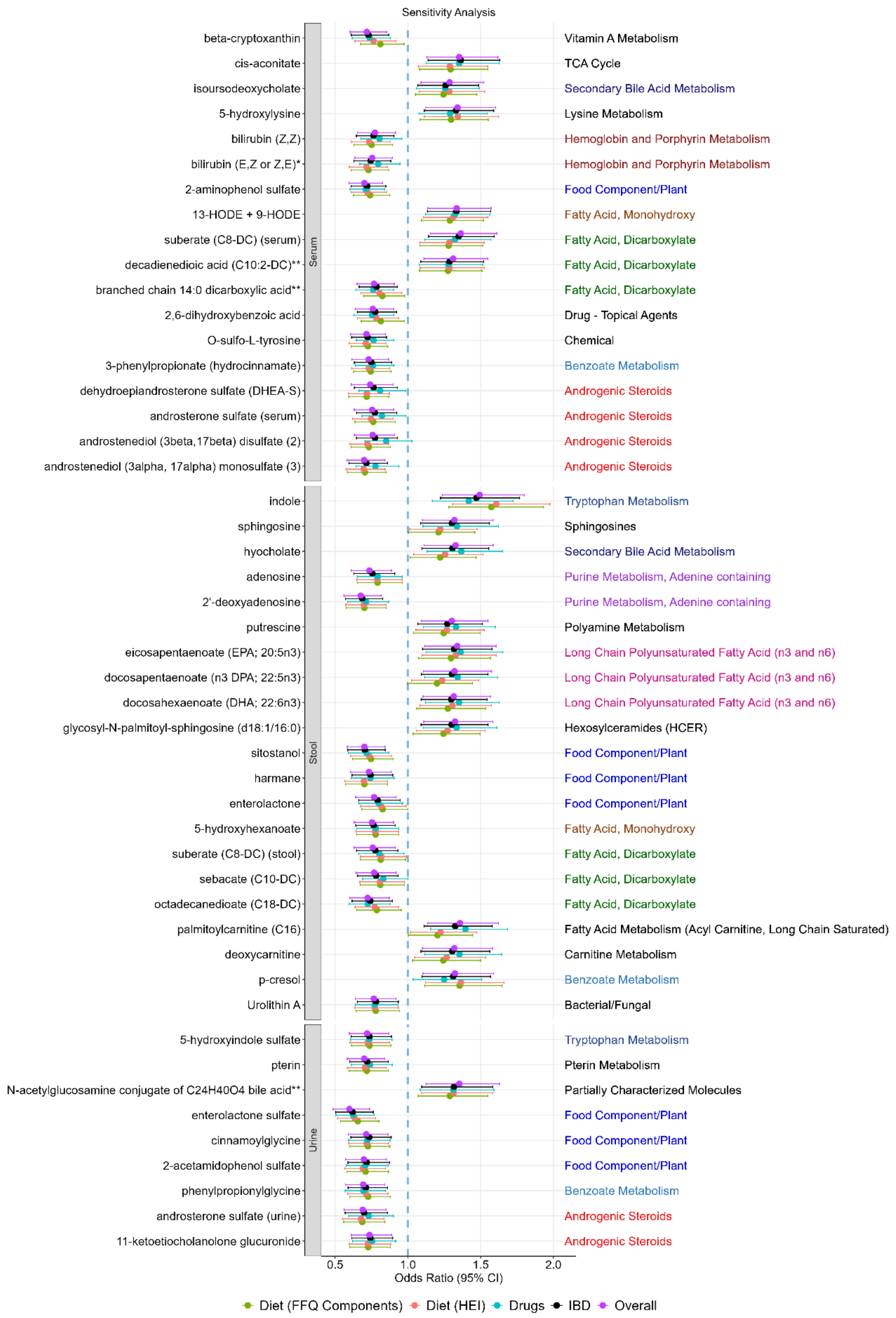

Supplement: Supplementary file 1 [file metabolites-15-00121-s001.zip › metabolites-3346152-supplementary.pdf]
